# U-Net Optimization for Hyperreflective Foci Segmentation in Retinal OCT

**DOI:** 10.3390/diagnostics16060853

**Published:** 2026-03-13

**Authors:** Pavithra Kodiyalbail Chakrapani, Preetham Kumar, Sulatha Venkataraya Bhandary, Geetha Maiya, Shailaja Shenoy, Steven Fernandes, Prakhar Choudhary

**Affiliations:** 1 Manipal Institute of Technology, Manipal Academy of Higher Education, Manipal 576104, India; pavithraforu@gmail.com (P.K.C.); geetha.maiya@manipal.edu (G.M.); prakharchoudhary09@gmail.com (P.C.); 2 Department of Ophthalmology, Kasturba Medical College, Manipal Academy of Higher Education, Manipal 576104, India; sulatha.bhandary@manipal.edu (S.V.B.); shailaja.s@manipal.edu (S.S.); 3 Department of Computer Science, Design and Journalism, Creighton University, Omaha, NE 68187, USA

**Keywords:** Hyperreflective Retinal Foci, segmentation, biomarker, diabetic macular edema, optical coherence tomography, deep learning, health, disease, diabetes

## Abstract

**Background/Objectives:** Hyperreflective foci (HRF) are supportive optical coherence tomography (OCT) imaging biomarkers that have been examined for their association with disease progression and severity in various retinal disorders. The accurate identification and segmentation of these tiny structures of lipid extravasation remain complicated because of their small size, class imbalance, similarity in the reflectivity patterns with the surrounding structures and imaging artifacts. While U-Net-based models have promised exceptional results for medical image segmentation, optimal architectural settings and suitable preprocessing methods for HRF detection remain unclear. **Methods:** This research assessed optimal settings for U-Net-based models for HRF segmentation by evaluating standard U-Net and attention U-Net under different preprocessing regimes. Attention U-Net employed Z-score normalization and contrast-limited adaptive histogram equalization (CLAHE) enhancement with soft dice loss. The standard U-Net was trained on OCT images with CLAHE using focal Tversky loss. A total of 435 fovea-centered OCT B scans with the corresponding, consensus-annotated HRF masks were utilized for this research. **Results:** The standard U-Net outperformed attention U-Net with a dice score of 0.5207, an AUC of 0.8411, and a recall of 0.6439 on raw OCT images. The attention U-Net with preprocessing (dice: 0.5033, AUC: 0.6987, recall: 0.5391) demonstrated satisfactory performance. The results showed that the U-Net model with CLAHE and focal Tversky loss improved recall by 19.4% relative to the attention U-Net, and this corresponds roughly to a 23% relative decline in false negatives. This indicates increased sensitivity in identifying HRF regions. **Conclusions:** The best-performing configuration using U-Net-based architectures for segmentation of HRFs combines the standard U-Net model with CLAHE and focal Tversky loss for handling class imbalance. This approach yields relatively higher sensitivity, indicating that the standard U-Net model delivers a simple and robust framework for automated HRF segmentation on the evaluated dataset, promising further validation in broader clinical datasets.

## 1. Introduction

The most prevailing microvascular complication of diabetes mellitus is the retinal disease diabetic retinopathy (DR). DR threatens retinal health and remains the main cause of preventable blindness and vision loss in people of working age in developed countries. Diabetes is becoming more and more common globally. The statistics report that 592 million people will acquire the disease by 2035 [1]. The vision-threatening complications will increase proportionally with the presenceof DR, leading to major public health and socioeconomic concerns. Among the various warning signs of DR, diabetic macular edema (DME) remains the main risk factor of vision loss in diabetic patients [2]. DME is represented by the buildup of fluid in the macula as a result of the blood–retinal barrier breaking down. This is caused by a number of intricate pathophysiological processes, such as oxidative stress caused by chronic hyperglycemia, inflammation, upregulation of vascular endothelial growth factor (VEGF), and progressive microvascular damage [2,3]. DME may appear at any point in the evolution of DR and has an important impact on central visual acuity, which in turn affects patients’ ability to function and their quality of life [2,4].

The advent of spectral-domain OCT (SD-OCT) has comprehensively enhanced the way in which retinal diseases like DME are detected, monitored, and treated [5]. SD-OCT is a non-invasive, very high resolution imaging method that provides detailed 3D cross-sectional views of retinal tissues [5,6]. Ophthalmologists are able to detect tiny morphological alterations within the layers of the retina, quantify the thickness of the retina, and detect other pathological features that are not identifiable with conventional imaging methods. OCT offers very high quality retinal scans along with enhanced speed of image acquisition. OCT has become the gold standard for DME evaluation, providing various advantages like not requiring the injection of dye, being non-contact, and allowing for the quantification of retinal abnormalities that cannot be achieved by conventional imaging modalities [7].

### 1.1. Hyperreflective Retinal Foci (HRF)

**Figure 1:**
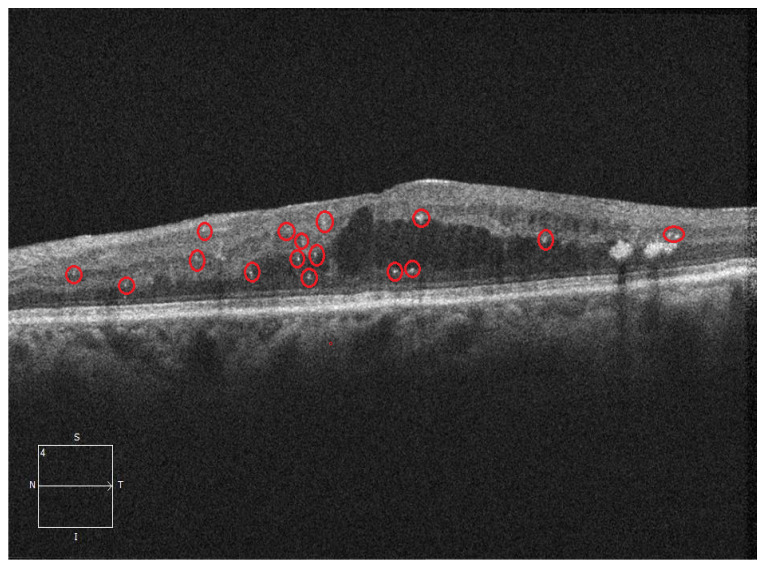
OCT B-scan depicting the presence of HRF. Selected HRFs are highlighted with red circles to facilitate visual identification.

OCT images give out a lot of information regarding the abnormalities within the retinal structures. As a valuable finding in OCT imaging, HRF has attracted a lot of attention because of its potential role as a biomarker in a variety of retinal disorders [8]. HRF are well-defined, tiny, punctate lesions resembling small white-colored dots on an OCT image that have a reflectivity similar to or greater than that of the retinal pigment epithelium (RPE) layer [9]. HRFs are typically very tiny structures with dimensions approximately close to 20–40 μm in diameter [10]. Most of the time, they are spread over various layers of the retina from the inner limiting membrane to the RPE. The presence of HRF, the region where they appear, and their quantity are associated with the severity of various retinal diseases, guiding significant clinical decisions [6,11,12]. Multiple explanations have been put forward in the literature, but the pathophysiological cause of HRF is still being researched. According to the theory by Bolz et al. [9], HRF are structural signs of lipid deposition following damage to the blood–retinal barrier that are precursors of clinically apparent hard exudates. Some journal papers have reported that HRFs are linked with stimulated microglial cells, which are the resident immune cells of the retina. These cells transit and alter structurally as a result of retinal injury [6,11]. Figure 1 depicts the existence of HRF in retinal OCT images.

Recent research reports that the presence and amount of HRFs tend to have a very close correlation with the other OCT biomarkers of DME leading to poor vision. The existence of HRFs, especially in the outer retinal regions, is linked to disruption of the external limiting membrane (ELM) and ellipsoid zone (EZ), leading to diminished visual acuity in DME patients. Even with structural recovery with anti-VEGF treatment, patients with higher HRF counts demonstrate a low rate of visual acuity improvement. This shows that HRF might cause irreversible damage to the photoreceptor or chronic inflammatory responses that prevent recovery of vision [12].

### 1.2. Challenges in HRF Detection and Significance of Automatic Segmentation

Despite the therapeutic importance of HRFs, there are a number of challenges involved in the identification and assessment of HRFs in the clinical domain. Lei et al. proposed that an HRF has a size of 20 to 50 μm, exhibits high reflective intensity, and lacks back shadowing. In addition, evaluating complete OCT volumes containing multiple B-scans in clinical OCT datasets is tedious and impractical in medical facilities [13]. While HRFs are readily identifiable during routine clinical OCT examination, supervised deep learning approaches require manually delineated pixel-level ground-truth segmentation masks for training. Even though there is agreement on the presence of HRFs during manual annotations, there may be some inter-observer variability during boundary annotation, particularly for small or low-contrast HRFs where exact pixel-wise delineation can be subjective [14]. HRFs often have a very small size of 20–40 μm, and they have variable contrast due to the characteristics of the surrounding tissue. HRF detection involves potential conflicts with the remaining hyperreflective structures, like hard exudates, retinal vessels, or imaging distortions [6,15].

### 1.3. Motivation and Contributions

The uneven distribution, inter-observer variability, small dimensions, and scarcity of annotated datasets make it challenging to accurately segment HRF in OCT images. The application of existing research in actual clinical settings is limited since they frequently rely on region-level detection and big datasets [14]. These limitations have encouraged the design of computer-aided segmentation models for HRF detection, involving deep learning (DL) and enhanced image processing techniques. Artificial intelligence-based DL methods, especially those involving convolutional neural networks (CNNs), have yielded impressive performance in the segmentation and detection of pathological lesions in retinal OCT B scans, including the analysis of other medical images [16]. The main contributions of this research are as follows:

- A systematic evaluation of U-Net-based architectures for pixel-level segmentation of HRFs using a small and carefully annotated OCT dataset.
- An analysis of model complexity versus generalization performance, highlighting the effectiveness of simpler architectures for limited-data OCT imaging scenarios.
- The use of an ethically approved, fully anonymized OCT dataset with consensus-based manual annotations, ensuring data integrity.

The proposed research assesses the performance of U-Net and attention U-Net models for automated segmentation of HRF from OCT B scans of DME patients.

## 2. Related Work

The following studies collectively demonstrate the use of AI for identification of HRFs from OCT images in the literature. Mokhtari et al. [17] conducted an initial study with an automatic detection of HRF from OCT scans employing a Morphological Component Analysis method. They aimed at separating curve singularities (retinal layers) from point singularities (HRF) using curvelet and wavelet dictionaries to differentiate foci from retinal layers. In an attempt to identify predictive biomarkers for DR severity, Niu et al. [18] used a support vector machine (SVM) model with leave-one-out cross-validation. They performed a multi-modal analysis using color fundus and OCT images, and the research revealed that an HRF has a greater axial altitude (an average of 69.6 μm in proliferative DR and 53.5 μm in non-proliferative DR) that exists along the borders of ONL-OPL layers and corresponds to hard exudates that are clinically visible. Katona et al. [19] developed approaches for HRF identification using DL-based techniques like CNNs, ANNs, and DRNs employing OCT images of AMD patients and achieved correlation coefficients of 0.845–0.862. They used manually annotated OCT images by the ophthalmologists. They could report correlation coefficients greater than 0.845 with CNN-based implementation. Their results demonstrated segmentation performance comparable to reported inter-observer agreement levels in the literature.

Okuwobi et al. [14] combined graph search and random forest classifier for layer segmentation along with the automated grow-cut algorithm for HRF segmentation. Transfer learning strategies with InceptionResNet50, ResNet50, and Inception-v3 architecture were employed to automatically detect HRFs and other biomarkers in OCT images of AMD patients [20]. Varga et al. [21] developed a framework that integrated multiple neural networks, image processing, and feature extraction for HRF segmentation, with FCN yielding the best performance. FCN could achieve a dice value of 0.798 for 3-pixel tolerance on test data. Yu et al. [22] proposed modified GoogleNet and ResNet CNN frameworks with strides and small kernel sizes to handle classification of HRFs based on small OCT patches. Okuwobi et al. [23] combined morphological reconstruction-based clustering for generating ROI with component tree-based region extraction for estimating HRF with the elimination of the layer segmentation step. An ensemble-based DL approach leveraging modified Squeeze-and-Excitation blocks (SE-block), U-Net, a VGG-16 variant, and a Pix2Pix GAN was utilized to segment retinal fluids and HRFs from OCT, with the ensemble method outperforming the individual baseline approaches [24]. Xie et al. [25] introduced an enhanced 3D U-Net model for fast and automated segmentation of HRFs utilizing slice-based dilated convolutions within the encoder path to extract a broad range of spatial data across OCT volumes. They leveraged two-channel input to the model with the combination of enhanced and denoised B-scans and demonstrated superior results in low-contrast images. Huang et al. [26] and Yao et al. [27] employed improved U-shaped CNNs (multi-scale convolution modules and channel attention modules) and SANet (Self-Adaptive Network) based on U-Net architecture based on dual residual modules for the segmentation of HRFs.

Midena et al. [28] employed open-source software ImageJ for the semi-automatic detection of small HRFs (size < 30 μm) that are identified as representing microglial cells as inflammatory biomarkers in diabetic retinopathy (DR). Goel et al. [29] incorporated an OCT patch-based learning approach for enhanced performance by improving computational focus on HRF pixels for their segmentation. They used ResNet50 for classifying OCT patches and U-Net with a ResNet34 backbone for segmentation. Their research reported that identification of the region of interest can automatically enhance the HRF segmentation performance. Recent developments in computerized HRF detection, including Zhou et al.’s [30] method based on optical attenuation coefficient for depth-aware quantification and visualization, Yao et al.’s [31] GD-Net architecture for joint multi-class segmentation of microglia and hard exudates, and Schmidt et al.’s [32] blob detection with CNN-based identification of HRFs on the outer nuclear layer, demonstrate the usefulness of integrating conventional image processing with DL-based architectures.

Midena et al. [6] proposed an AI algorithm for retinal layer and fluid assessment along with HRF quantification, reporting high consistency between automated and manual evaluations. A lightweight DBR model for the detection of over three retinal diseases is developed by Wei et al. [33], and achieved a dice of 0.8076 with an inference time of 57 ms per image, demonstrating the ability for medical deployment. A hierarchical feature extraction-based RC-Net model with region-level attention was validated by Zhang et al. [34], and distinguished small-scale HRFs from complex backgrounds. A texture-based labeling framework along with conventional image processing and SVM was leveraged by Monemian et al. [35], who used intensity gradient analysis for HRF identification. Ying et al. [16] published a systematic review including most of the DL-based HRF segmentation models in the literature concerning various retinal disorders by consolidating the advantages and limitations along with image quality dependencies, dataset size, varied patient population, etc.

Wang et al. [36] utilized MUSE-Net through the multi-dimensional semantic improvement, integrating adjacent slice information for inter-slice context and dynamic region information for the intra-slice context for HRF segmentation. The results were validated on two datasets with clinical treatment monitoring examples. Among recent deep learning approaches, Li et al. [13,37] proposed U-Net and attention U-Net-based frameworks for automated HRF segmentation in OCT images. To improve the localization of tiny and higher-intensity HRF regions, the authors used attention mechanisms and multi-scale feature fusion. They utilized a small dataset of 170 OCT B-scans acquired from multiple patients for model evaluation. With five-fold cross-validation, they reported comparatively balanced sensitivity and precision, with dice values exceeding those tabulated in several prior studies. The details of all the publications in the literature, including the datasets and results, are listed in Table 1. The majority of the segmentation-based studies have assessed various architectures under identical training configurations and fixed input dimensions, which might lead to loss of small HRF regions [13,37]. This research aims to assess the interaction between model complexity and loss function selection for HRF segmentation. Rather than doing a direct architectural comparison, the aim of this study is to identify appropriate preprocessing and training parameters for U-Net-based architectures to perform reliable HRF segmentation from OCT pictures. This study attempts to explore segmentation of HRFs using OCT B-scans at their original resolution to retain fine-grained structural details that may be eliminated by traditional resizing methods. The standard U-Net was trained on raw OCT images without using any preprocessing, but the attention U-Net needed CLAHE enhancement and Z-score normalization to obtain stable and improved segmentation performance.

## 3. Materials and Methods

### 3.1. Dataset Description

The OCT image dataset employed for this research was gathered from patients identified with DME from the Department of Ophthalmology, Kasturba Medical College (KMC), Manipal Academy of Higher Education, Manipal, India. We obtained approval from Kasturba Medical College and Kasturba Hospital Institutional Ethics Committee, Manipal, with the approval code IEC1-287/2022. Further, all the procedures were performed in compliance with the principles of the Declaration of Helsinki and the ethical guidelines stated by the ethical committee. The KMC dataset had completely anonymized, original, fovea-centered OCT B-scans of 435 adult male and female patients (age > 18 years) diagnosed with DME associated with type 2 diabetes mellitus, mostly from Karnataka, India. These images were acquired through a Zeiss Cirrus HD OCT 5000 (Carl Zeiss Meditec AG, Jena, Germany) imaging device deployed at KMC, Manipal.

### 3.2. Consensus Annotation and Inter-Observer Variability

**Figure 2:**
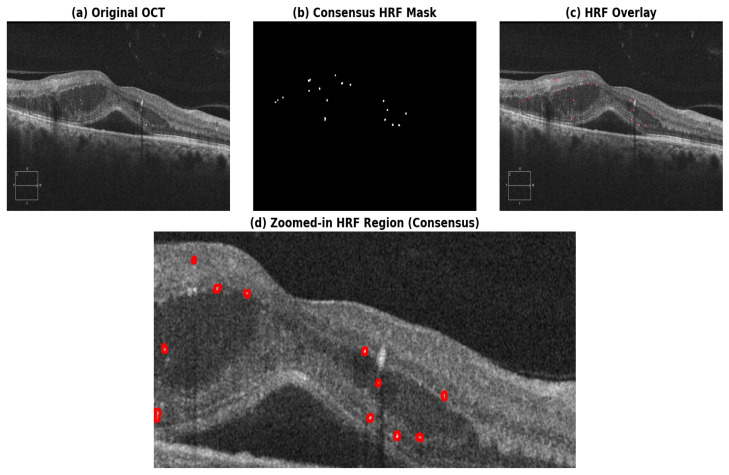
Visualization of HRF annotations based on consensus to minimize inter-observer variability. (**a**) Original OCT B-scan; (**b**) annotated consensus HRF binary mask; (**c**) overlay of HRFs on the OCT image, with HRFs marked by red circles; and (**d**) magnified view illustrating the small and sparse distribution of HRFs, also marked with red circles.

HRFs are small, punctate structures in OCT B-scans that are challenging to delineate manually and are subject to inter-observer variability, as their appearance overlaps with speckle noise and other hyperreflective retinal attributes more often [13]. To eliminate inconsistencies in the annotations and enhance the reliability of ground-truth labels, the doctors adopted a consensus-based annotation protocol for the research. The OCT images were jointly annotated by two ophthalmologists following a consensus-based review process. The reviewers evaluated the images together, rather than independently, using a cloud-based annotation platform (Apeer Annotate) [39]. The disagreements with respect to the presence/absence, number of pixels, and their boundaries were resolved through discussion to achieve mutual consensus. All the HRF annotation masks were exported and downloaded in .ome.tiff format. This joint annotation method guaranteed that the ambiguous HRF structures were adequately examined, thereby eliminating individual bias and avoiding inter-observer conflicts among the final HRF annotations. The Figure 2 shows the final consensus annotation of a sample OCT image. The finalized consensus masks were considered as the reference standard for training and assessing the proposed U-Net-based models.

### 3.3. Dataset Splitting and Augmentation

Ground truth labels were obtained in scientific TIFF format (.ome.tiff), and they were processed with the tiffle package to adequately handle multi-channel structures and metadata. Among the multiple channels in the masks, the first channel was extracted for binary segmentation. Different preprocessing strategies were utilized to report the best setting for each of the models. OCT images were preprocessed while maintaining their natural resolution for both the models, standard and attention U-Net. Processing the images at their original resolution helps to preserve the fine-grained HRF features that might be lost during traditional downsampling or resizing. With standard U-Net, images were preprocessed with CLAHE. But, for the attention U-Net, we employed CLAHE followed by Z-score normalization to improve the contrast of the images and normalize intensity differences among them. OpenCV was used to process the input images to convert them to the RGB colour space. Mask values were binarized with a zero threshold, where a pixel having a value greater than zero was categorized as a positive class (HRF), and a pixel with a value equal to zero was classified as a negative class (background). The whole dataset was randomly split using a fixed random seed (seed = 42) to ensure reproducibility, with 70% (304 scans), 15% (65 scans), and 15% (66 scans) allocated to the training, validation, and testing sets, respectively. Data augmentation was applied to the training data used with the standard U-Net to increase the number of samples and improve model generalization. Random rotations, scaling, horizontal and vertical flips, and intensity augmentations were applied to the training data before training using the Albumentations library.

### 3.4. Model Architectures

**Figure 3:**
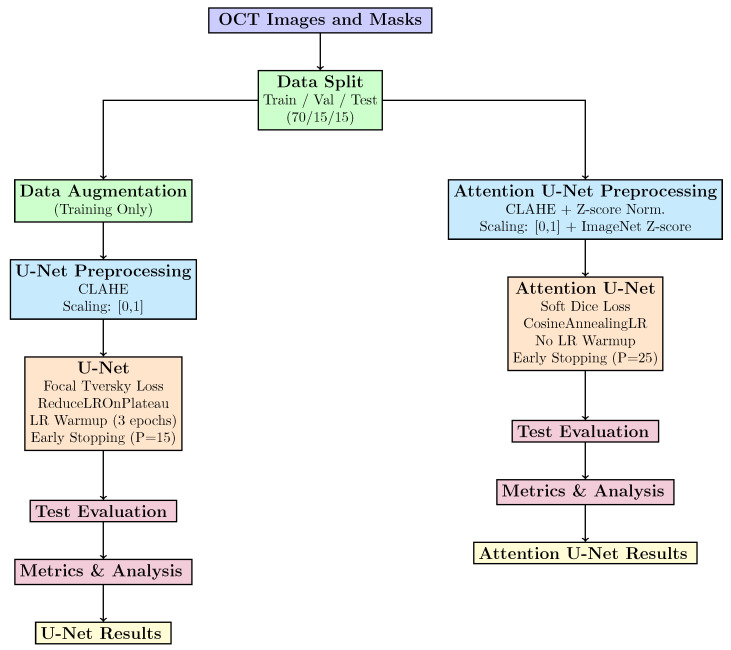
Overall methodology adopted for HRF segmentation using U-Net and attention U-Net architectures with independent evaluation pipelines.

The conventional encoder–decoder U-Net architecture with a skip connection was used in the research. The encoder has four downsampling blocks, each consisting of two convolutional layers (3 × 3 kernels) and batch normalization followed by ReLU activation and 2 × 2 maxpooling for downsampling. The initial number of filters used was 64, doubling at each higher level from 64 to 128, 256, and 512. Rather than employing bilinear upsampling to retain learnable parameters, the decoder mirrored the encoder structure with four upsampling blocks using transposed convolutions (stride 2, kernel 2 × 2). At the final layer, 1 × 1 convolutions were used to produce a single-channel output, followed by sigmoid activation. The model’s trainable parameters were approximately 31 M, enabling it to be efficient for real-world deployment. Further, an attempt was made to evaluate the attention U-Net that enhanced the standard U-Net by utilizing attention gates at each skip connection stage. The spatial attention coefficients, which weighted the features of the encoder prior to concatenation with the decoder features, were computed by the attention gates to improve the model by highlighting important features while suppressing the irrelevant details significant for segmentation. The attention-based approach increased the parameters by approximately 10–15% compared to the standard U-Net. To provide for a fair comparison, all other factors, including the number of filters, encoder depth, and decoder structure, were kept identical to those of the standard U-Net.The overall methodology involved in the research is shown in Figure 3.

The following Equation (1) defines the Tversky loss function [40], which is particularly suitable for segmentation tasks with severe class imbalances.

(1) $$L_{\mathrm{Tversky}}\left(Y,\hat{Y}\right)=1-\mathrm{TI}=1-\frac{\sum_{i=1}^{HW}Y_{i}\cdot {\hat{Y}}_{i}+\epsilon }{\sum_{i=1}^{HW}Y_{i}\cdot {\hat{Y}}_{i}+\alpha \sum_{i=1}^{HW}\left(1-Y_{i}\right)\cdot {\hat{Y}}_{i}+\beta \sum_{i=1}^{HW}Y_{i}\cdot \left(1-{\hat{Y}}_{i}\right)+\epsilon },$$

where $Y_{i}\in \left\{0,1\right\}$ and ${\hat{Y}}_{i}\in \left[0,1\right]$ denote the ground truth and predicted label of the *i*-th pixel, respectively, and HW represents the total number of pixels in the image. The summation is performed over all pixels. The terms correspond to true positives (TPs), false positives (FPs), and false negatives (FNs), respectively. The parameters α and β control the relative penalty assigned to FPs and FNs, while ϵ is a small constant introduced for numerical stability. The quantity TI denotes the Tversky index. Additionally, to emphasize the hard-to-segment pixels, the focal Tversky loss is defined as

(2) $$L_{\mathrm{FT}}\left(Y,\hat{Y}\right)={\left(1-\mathrm{TI}\right)}^{\frac{1}{\gamma }},$$

where γ∈[1,3] is a focusing parameter that controls the degree to which misclassified pixels are emphasized, with larger values of γ placing greater emphasis on false negatives.

An important aspect of our research design was the usage of two different loss functions, adhering to the architectural characteristics of each model and the pixel-wise class imbalance that is present in HRF regions. The standard U-Net was trained with focal Tversky loss (Equation (2)) with the hyperparameters α = 0.3, β = 0.7, and γ = 4/3, which is designed specifically to manage severe class imbalance by penalizing the false negatives [40]. α and β control the penalty for false positives and false negatives, respectively. In contrast to this, a soft dice loss that computes the overlap between the ground truth and predicted masks to optimize the dice coefficient is used with the attention-U-Net model. L2 regularization of 0.0001 with the Adam optimizer with an initial learning rate of 0.001 is used by both the models to prevent overfitting. Additionally, the standard U-Net version employed an initial three-epoch warm-up period for the learning rate. Two different learning rate schedulers were used by each of the models, as shown in Figure 3. Two different strategies for scheduling were used to reflect typical training procedures used with each model in the literature.

Each experiment was carried out using PyTorch (version 2.9.1) on a high-performance computing (HPC) platform featuring NVIDIA A100 80 GB PCIe GPUs with CUDA 12.8. To guarantee consistent training and effective memory use, the batch size was fixed to 4 images. Based on the validation dice score, the models were trained for 100 epochs with an early stopping patience of 15 epochs for the conventional U-Net and 25 epochs for the attention U-Net, and the best models were saved using checkpoints.

## 4. Results

**Figure 4:**
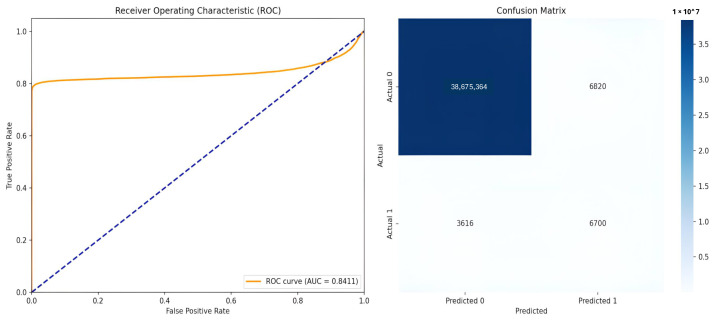
ROC curve and confusion matrix generated by the standard U-Net on the test set. The blue dotted diagonal line represents the performance of a random classifier.

**Figure 5:**
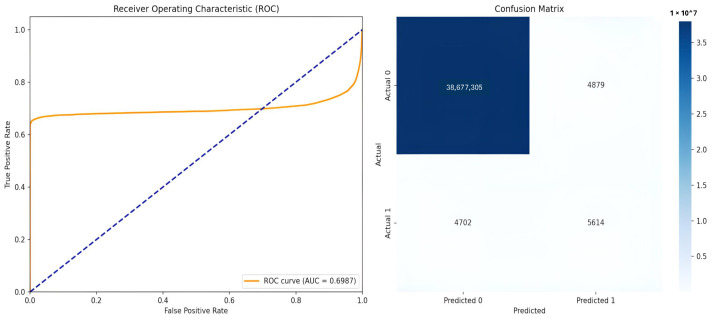
ROC curve and confusion matrix generated by the attention U-Net on the test set.

Different performance measures reported at the pixel level across all test images were used to analyze the model’s efficiency. The Dice Similarity Coefficient, which measures the overlap between ground truth and predicted masks; Intersection over Union (IoU/Jaccard Index); Precision (positive predictive value); Recall (sensitivity/true positive rate); F1 Score; and Specificity were reported. Further, we reported the area under the receiver operating characteristic curve (ROC-AUC) before thresholding, using predicted probabilities. Confusion matrices were computed to demonstrate true positives (TPs), true negatives (TNs), false positives (FPs), and false negatives (FNs) across all pixels in the test set. Table 2 demonstrates the values obtained for the performance measures with the U-Net and attention U-Net models. Figure 4 and Figure 5 exhibit the ROC curves and the confusion matrices generated by the standard U-Net and attention U-Net, respectively.

**Figure 6:**
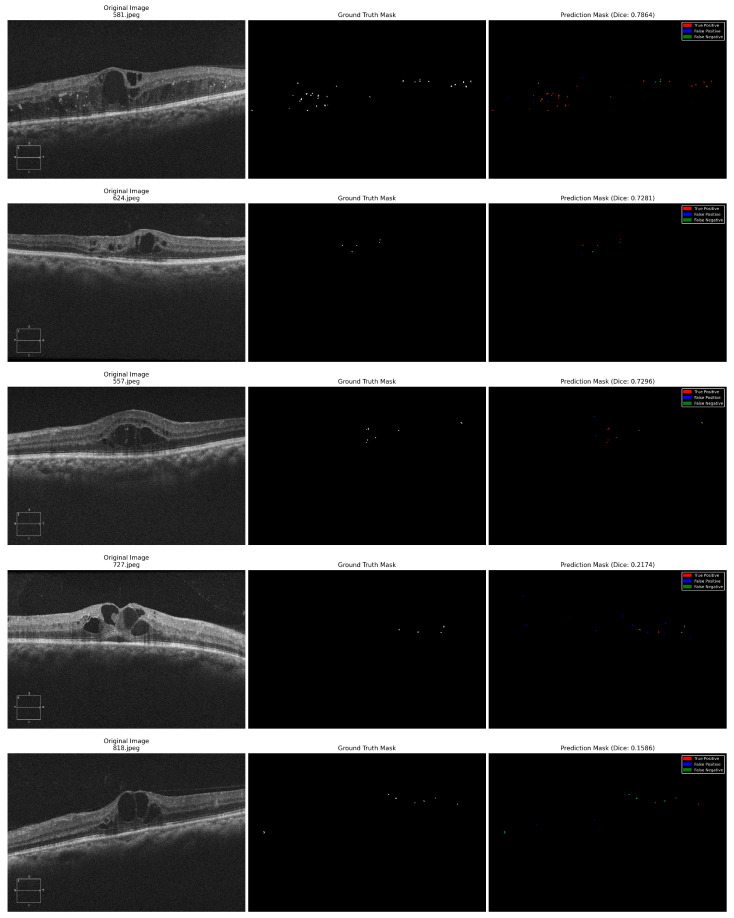
Standard U-Net HRF segmentation results for five OCT images with the original OCT image, ground truth HRF mask, and predicted HRF mask combined into a single composite image. The first four examples show effective detection of HRFs. The next two examples show challenging cases with lower dice scores, highlighting typical failure modes including missed HRFs in poor-contrast regions and false positives in distorted retinal structure. Different colors have been used to denote true positives (red), false positives (blue), and false negatives (green).

**Figure 7:**
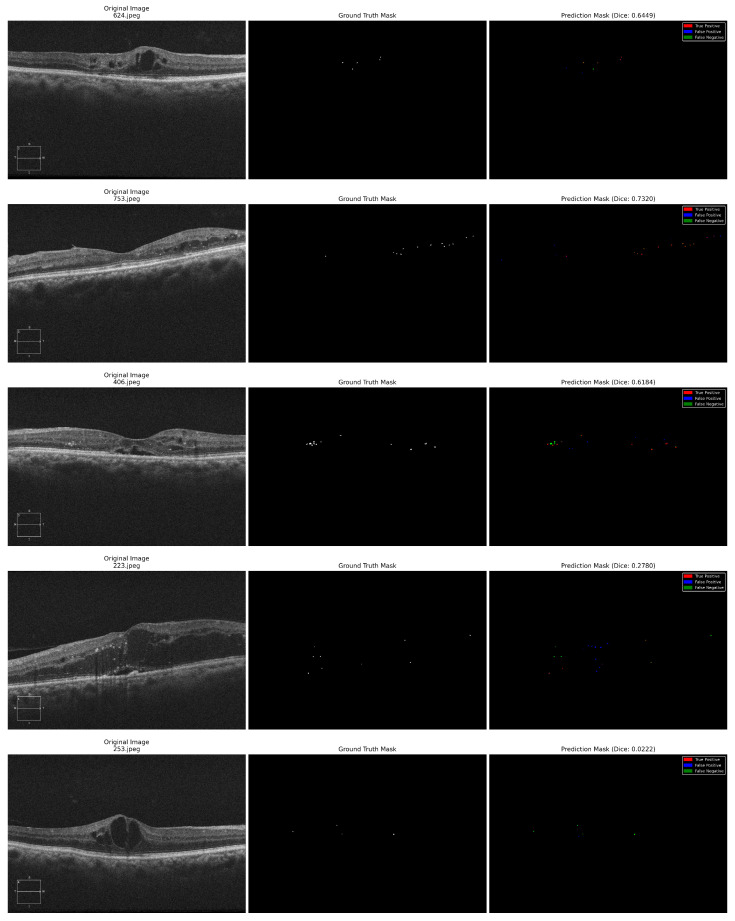
Attention U-Net HRF segmentation results for five OCT images with the original OCT image, ground truth HRF mask, and predicted HRF mask combined into a single composite image. The first four examples show effective detection of HRFs. The next two examples show challenging cases with lower dice scores, highlighting typical failure modes including missed HRFs in poor-contrast regions and false positives in distorted retinal structure. Different colors have been used to denote true positives (red), false positives (blue), and false negatives (green).

The standard U-Net achieved a dice score of 0.5207, an IoU of 0.3655, and a ROC-AUC of 0.8411 on the independent test set of OCT images. The model further attained a precision of 0.4800, recall of 0.6439, F1-score of 0.5184, and a high specificity of 0.9998, indicating effective suppression of false positives at the pixel level. There is a slight reduction in metric values for the attention U-Net (dice score of 0.5033, IoU of 0.3515, and a ROC-AUC of 0.6987), suggesting that the incorporation of attention mechanisms did not yield consistent performance gains for HRF segmentation in the evaluated test set. Figure 6 and Figure 7 show the predicted HRF pixels by the U-Net and attention U-Net models, respectively.

## 5. Discussion

For HRF segmentation from OCT images, the present research demonstrates that the U-Net model outperformed the attention U-Net, achieving a dice coefficient of 0.5207 compared to 0.5033, corresponding to a 3.46% improvement in segmentation accuracy. Importantly, the conventional U-Net exhibited superior discrimination capability, attaining an AUC of 0.8411 versus 0.6987 for the attention U-Net, reflecting a 20.38% enhancement in the model’s ability to distinguish between background and HRF pixels across varying threshold values. These results raise questions about the conventional assumption that highly complex models with attention mechanisms provide high performance in medical image segmentation. The use of focal Tversky loss, which is specifically designed to address severe class imbalance by asymmetrically penalizing false negatives, along with the simpler architecture of the model, enables better generalization when training data are limited.It demonstrated more stable training without the additional optimization burden enforced by attention gates, which are the major contributing aspects to the superior performance of the standard U-Net.

To take advantage of a relationship between the spatial features of the loss function and the attention mechanism, we adopted soft dice loss for the attention U-Net. Soft dice loss optimizes the precise spatial overlap of these accurate predictions, as the attention U-Net’s gating mechanism effectively filters features to concentrate on important areas (HRF spots) and suppress noise from the background. The dominance of the background pixels often impacts pixel-wise losses (including cross-entropy) for tiny object segmentation tasks like HRF detection. This is lowered by soft dice, which makes sure the model aims for accurate border outlining instead of just high pixel-wise accuracy by penalizing mismatches in the foreground overlap. We noticed that this combination improved the results of the attention U-Net more than the focal Tversky loss.

The reason for not using identical settings for both models is that we aimed for the optimization of each model for maximum performance rather than focusing on the conduction of a rigorous component-wise ablation analysis within the computational resources and dataset that were available. We first evaluated the attention U-Net using the standard U-Net’s configuration (focal Tversky + minimal preprocessing) but saw suboptimal convergence. As the gating mechanism used in the attention U-Net is affected by the input feature magnitude distributions, the Z-score normalization was useful in the stabilization of attention coefficients. In the same way, soft dice loss was found to be better than focal Tversky loss through experiments in separating the HRFs from the background. Thus, we include the best-performing configurations for each model to illustrate their peak performance under our resource and data constraints.

When analyzing the results of the two models, a distinct precision–recall trade-off was observed. The standard U-Net demonstrated better sensitivity in segmenting HRF pixels with a low possibility of overlooking actual erroneous regions with a recall of 64.39%. The attention U-Net exhibited a more conservative prediction pattern, which lowered false positives by predicting HRF pixels exclusively with high confidence, as seen by its higher precision of 52.65%. This difference in the results indicates distinct model behaviors. The attention U-Net favored precision, and the standard U-Net prioritized sensitivity, which is more important and desirable in clinical HRF detection circumstances where false negatives might lead to disease severity underestimation. The findings demonstrate that the choice of loss function seems more difficult than dealing with the complexity of the model for managing pixel-wise class imbalance in the medical domain. Focal Tversky loss with the standard U-Net yielded better results than soft dice loss with the attention U-Net, signifying that the ability of addressing class imbalance with asymmetric weighting makes up for the absence of attention mechanisms. Additional research is required to figure out how loss functions affect simple versus complex models in various ways, especially if attention mechanisms work better when combined with loss functions created especially for complex networks.

Very recent CNN-based approaches reported in Table 1 have attained higher dice values, even exceeding 0.70 [21,25,33,35]. But these publications typically used different method formulations, substantially larger datasets, tuning of the methods specific to devices, and they differ from pixel-level segmentation of sparsely distributed HRFs. For example, Wei et al. [33] and Varga et al. [21] utilized thousands of B-scans for more robust training, while Yao et al. [27] employed attention-based architectures under monitored image capturing conditions. In contrast, multiple studies using limited datasets have exhibited moderate results with dice scores fluctuating between 44% and 66% [22,24,26], demonstrating the inbuilt issues of HRF segmentation. All these findings seem consistent with the trends in the observed results of the proposed study. In contrast to previous research, this work examines the relationship between architectural complexity and generalization performance by methodically evaluating U-Net-based designs using a small, completely anonymized OCT dataset with consensus-based annotations. The reported advantages of simple architectures match the common trend in Table 1, where highly complex models were not able to provide exceptional results in limited data settings.

Given the small medical dataset exhibiting severe class imbalance, the poor performance shown by Attention U-Net in our study highlights the significant limitations of attention mechanisms. Attention gates increase the number of parameters by approximately 10–15% (34 M from 31 M). Although this difference is small, it could lead to overfitting when training data is scarce, in contrast to the conventional U-Net. The higher recall provided by the standard U-Net indicates an intentional architectural choice to reduce missed HRFs. Although decreased precision might result in increased false positives, the approach is meant for clinician-guided review rather than independent decision-making. By retaining the original resolution, we ensured that tiny HRFs were not lost during downsampling and that it is easier for the models to learn to handle natural variations in image quality in clinical practice. U-Net may be adapted to help process raw OCT data with varying intensity distributions and variable contrast. Direct comparison of this research with previously published studies seems difficult because of the differences in model design, annotation granularity, and dataset size, even though some researchers report higher dice scores. The proposed pixel-level segmentation under constrained data conditions demonstrates a more challenging scenario.

### 5.1. Failure Case Analysis

Two major reasons for failure restricting model output have been identified by analysis of representative failure case examples in Figure 6 (727.jpeg and 818.jpeg) and Figure 7 (223.jpeg and 253.jpeg). Low-contrast shadows (case 818.jpeg, dice: 0.1586 for U-Net) occurs when local picture quality is affected by posterior shadowing from surrounding cyst formations, resulting in a large number of false negatives as HRF becomes indistinguishable from background noise. Morphological distortion produces false positives when the hyperreflective edges of large cysts are incorrectly identified as HRF because of similar local intensity distributions (case 727.jpeg, Dice: 0.2174 for U-Net). In these challenging circumstances, U-Net and attention U-Net exhibited nearly identical failure patterns, suggesting that fundamental image quality issues are the main cause of performance degradation rather than architectural differences. According to these findings, automated image quality assessment might be used in clinical utilization to determine cases that need manual review. Future developments should focus on preprocessing improvements (contrast adjustment and denoising), as well as the incorporation of anatomical context to differentiate pathological HRF from structural artifacts.

### 5.2. Clinical Relevance and Applicability

Although routine clinical decision-making continues to rely on clinicians’ prompt qualitative interpretation of OCT scans, automated HRF segmentation currently serves mainly as a research-support tool. By generating pixel-level segmentation masks of HRFs, the proposed work enables objective and reproducible delineation of HRF regions, which would otherwise require time-intensive manual annotation. This supports consistent evaluation of larger OCT datasets in research workflows. More importantly, this research is intended to complement, not replace, routine clinical assessment. In the future, automated HRF quantification may assist doctors during patient follow-up by offering longitudinal comparisons across visits, enabling tracking of changes in HRF details over time. Automated HRF segmentation may enable consistent measurements over time and across hospitals, provided it is validated across various OCT machines.

### 5.3. Limitations and Future Directions

To clearly define the role and advantages of attention mechanisms, further research should perform thorough model evaluations with identical training settings. We admit that the dataset used for the study is small, which may not be sufficient to realize the benefits of using attention mechanisms for the models. We have not experimented with architectural changes like placement of attention gates and quantity, various attention gate designs, or hybrid methods that combine both aspects. A limitation of this research is that although the dataset of 435 scans is comparable to similar studies in the literature, it is not large enough for DL applications. Larger datasets would allow for the learning of more subtle features and a better assessment of model generalization to a variety of patient populations and imaging conditions, even though we used data augmentation and native resolution processing to maximize dataset utilization. Though dealt with by focal Tversky loss, the significant class imbalance, with HRF pixels making up less than 0.03% of total pixels, remains a fundamental challenge in this field. Future research should assess transfer learning from related retinal imaging tasks with larger labeled datasets, investigate semi-supervised methods for segmentation to use large volumes of unannotated OCT images, and perform multi-center validation on independent datasets from various OCT devices and clinical centers. The study demonstrates how simpler architectures can generalize more effectively than complex attention-based approaches when training data is limited. Larger and more diverse datasets are needed to effectively utilize attention mechanisms, even though overfitting was reduced by regularization and validation techniques. Developing models with larger datasets and optimal parameter will be given top priority in future endeavors. We did not perform a sensitivity analysis of the loss function weighting parameters (α,β,γ), as these were fixed to commonly used values reported in the prior literature to maintain experimental consistency. This design choice ensured an unbiased comparison across models. Future work will focus on conducting a comprehensive sensitivity analysis and exploring adaptive weighting strategies to assess the robustness of the proposed approach.

There is significant potential for improving model performance with the use of ensemble methods, multi-scale processing techniques, or semi-supervised learning utilizing unlabeled OCT data incorporating external validation and confounded architectural comparison. We could not perform cross-validation due to computational constraints associated with deep neural networks on high-resolution OCT images for 100 epochs per run. Another limitation is that the study did not use formal statistical significance testing. Hence, our future research will focus on statistical analysis of the results obtained through rigorous k-fold cross-validation, including numerous data splits to further demonstrate model robustness and statistical significance. Researchers can focus on the use of attention mechanisms suitable for small and sparse HRF features. Though this approach guaranteed highly valid and clinically consistent annotations, we acknowledge that the lack of quantifiable inter-observer agreement metrics is a downside. Future research may include independent annotations followed by consensus to enable proper analysis.

To assess robustness against scanner- and population-related domain shifts, future attempts may focus on multicenter validation utilizing bigger and heterogeneous OCT datasets that include various patient populations and OCT devices that use different image acquisition protocols. Because of the complete anonymization, detailed data about patient demography and severity of the disease were not available for analysis. While the dataset contained adult male and female patients with DME, subgroup-specific outcomes could not be analyzed. Clinically annotated datasets might be utilized during subsequent research studies to facilitate assessment that considers severity and background information. The augmentation methods used in the research are limited to basic geometric transformations to retain retinal morphology. More sophisticated HRF-specific augmentation techniques like elastic deformations and lesion-aware perturbations were not explored and are an important direction for future work. It is revealed from the failure case analysis that the cases with severe posterior shadowing or anatomical distortion exhibited degraded outcomes, which highlights the dependency of DL models on anatomical context and image quality. Future work might include more advanced image preprocessing methods along with the incorporation of structural priors.

## 6. Conclusions

This research attempts to find the best-performing configuration for U-Net-based architectures for computerized HRF detection through segmentation, including retinal OCT images. We performed a systematic analysis of model variants and preprocessing strategies to demonstrate that a standard U-Net with CLAHE provides superior results (Dice—0.5207, AUC—0.8411, Recall—0.6439) relative to the attention U-Net with CLAHE enhancement and z-score normalization (Dice—0.5033, AUC—0.6987, Recall—0.5391). The best-performing configuration was obtained through use of standard U-Net for efficient feature learning without attention, processing OCT images at their original resolution to preserve fine-grained HRF features, and the focal Tversky loss (α=0.3, β=0.7, and $\gamma =\frac{4}{3}$) function. These results reveal an important interaction between the preprocessing needs and the complexity of the architecture. The superior recall obtained by U-Net is vital and valuable clinically, as missing HRFs possesses higher diagnostic consequences than false positives. The proposed study provides vital insights that processing OCT images at their original resolution along with simpler models can outperform complex attention techniques with preprocessing of input. The findings may encourage further methodological advancement in this field and provide comparative performance evidence for automated HRF segmentation techniques. The recommended segmentation framework may facilitate research analysis and longitudinal monitoring rather than direct therapeutic use, and it is crucial to remember that HRF quantification alone is insufficient for clinical decision-making. Future evaluation involving various patient populations, data from multiple healthcare centers, and different OCT devices would help assess the generalizability of the proposed setting. Investigations using ensemble, transformer-based methods, and evaluation through clinical trials measuring diagnostic performance may be performed with real-world HRF screening initiatives.

## Figures and Tables

**Table 1:** Details of HRF Segmentation Methods in the Literature.

Reference (Year)	Dataset/Images	Acc. (%)	Sens. (%)	Spec. (%)	Prec. (%)	Recall (%)	Dice (%)
Mokhtari et al. [17] (2017)	496 B-scans	–	91.0	100.0	–	–	–
Niu et al. [18] (2017)	33 Patients	78.79	–	–	–	–	–
Katona et al. [19] (2018)	7 Patients	CC = 0.845 (CNN), 0.812 (inter-observer)					
Okuwobi et al. [14] (2018)	2560 B-scans	CC = 96.90% (PDR), 97.50% (DME)				–	62.3 (PDR), 63.8 (DME)
Saha et al. [20] (2019)	19,584 B-scans	89.0	78.0	100.0	–	–	–
Varga et al. [21] (2019)	911 B-scans	–	–	–	76.4–82.0	78.9–83.4	79.8–80.4
Yu et al. [22] (2019)	2304 B-scans	–	–	–	70.02 ± 14.38	65.95 ± 9.41	66.61 ± 9.16
Okuwobi et al. [23] (2019)	5120 B-scans	Dice: 69.70 (NPDR), 70.31 (PDR), 71.30 (DME)					
Sanchez et al. [24] (2020)	209 B-scans	–	–	–	–	–	44.37
Xie et al. [25] (2020)	4224 B-scans	–	–	–	72.68	68.89	70.73
Huang et al. [26] (2021)	2560 B-scans	–	–	–	70.02	65.95	46.00
Yao et al. [27] (2021)	112 B-scans	–	–	–	75.54	74.57	73.69
Midena et al. [28] (2021)	140 B-scans	Intraclass CC = 0.98					
Goel et al. [29] (2022)	3557 B-scans	–	–	–	75.0	–	57.0
Yao et al. [31] (2022)	202 B-scans	–	–	–	64.80	65.34	62.77
Zhou et al. [30] (2022)	49 eyes (24 test)	Significant correlation (*p* < 0.001) between automated and manual segmentation					
Schmidt et al. [32] (2023)	2596 B-scans	96.3	88.4	97.5	–	–	–
Midena et al. [6] (2023)	303 eyes	Kappa: 0.831 (SRF), 0.934 (ELM), 0.936 (EZ); ICC = 0.97 (HRF)					
Wei et al. [33] (2023)	3000 B-scans	–	–	–	75.54	–	80.76
Zhang et al. [34] (2024)	450 B-scans	–	78.36	–	75.34	75.29	62.27
Monemian et al. [35] (2024)	210 B-scans	96.0	86.0	98.0	82.0	–	84.0
Ying et al. [16] (2024)	Review	Comprehensive review of AI applications in HRF segmentation					
Wang et al. [36] (2024)	1120 images	–	76.7	–	75.1	–	72.4
Li et al. [13] (2025)	173 B-scans	–	72.90	–	66.12	–	66.84
Li et al. [37] (2025)	172 B-scans	–	66.66	–	67.10	–	63.79
Ying et al. [38] (2025)	39 eyes (DME)	Clinical validation of AI-based HRF segmentation and correlation with inflammatory cytokines					

Notes: A en dash (–) indicates that the metric was not reported in the original study. Accuracy reported by Niu et al. [18] corresponds to DR severity classification, not HRF segmentation. CC: correlation coefficient; ICC: intraclass correlation coefficient.

**Table 2:** Performance comparison of U-Net and attention U-Net for HRF segmentation.

Metric	U-Net	Attention U-Net
Dice Coefficient	0.5207	0.5033
Intersection over Union (IoU)	0.3655	0.3515
Precision	0.4800	0.5265
Recall (Sensitivity)	0.6439	0.5391
F1-score	0.5184	0.5007
Specificity	0.9998	0.9999
Jaccard Index	0.3910	0.3695
Area Under the Curve (AUC)	0.8411	0.6987

## Data Availability

The dataset generated and analyzed during this study is not publicly available due to ethical considerations. However, they can be obtained from the corresponding author upon reasonable request. Code for implementation can be found at: GitHub Repository (https://github.com/ai-research-2025/hrf-segmentation, GitHub, Accessed on: 5 March 2026).
